# Finger Tapping Task Activation vs. TMS Hotspot: Different Locations and Networks

**DOI:** 10.1007/s10548-019-00741-9

**Published:** 2019-11-06

**Authors:** Jue Wang, Hai-Jiang Meng, Gong-Jun Ji, Ying Jing, Hong-Xiao Wang, Xin-Ping Deng, Zi-Jian Feng, Na Zhao, Yu-Feng Zang, Jian Zhang

**Affiliations:** 1 School of Psychology, Shanghai University of Sport, Shanghai, 200438 China; 2 Department of Medical Psychology, Chaohu Clinical Medical College, Anhui Medical University, Hefei, 230032 China; 3 Collaborative Innovation Centre of Neuropsychiatric Disorder and Mental Health, Hefei, 230032 Anhui China; 4 Institutes of Psychological Sciences, Hangzhou Normal University, Hangzhou, 311121 China; 5 Zhejiang Key Laboratory for Research in Assessment of Cognitive Impairments, Hangzhou, 311121 China; 6 Center for Cognition and Brain Disorders and the Affiliated Hospital, Hangzhou Normal University, Hangzhou, 310015 China

**Keywords:** Finger tapping fMRI activation, TMS, Hotspot, Resting-state functional connectivity

## Abstract

Both functional magnetic resonance imaging (fMRI) and transcranial magnetic stimulation (TMS) have been used to non-invasively localize the human motor functional area. These locations can be clinically used as stimulation target of TMS treatment. However, it has been reported that the finger tapping fMRI activation and TMS hotspot were not well-overlapped. The aim of the current study was to measure the distance between the finger tapping fMRI activation and the TMS hotspot, and more importantly, to compare the network difference by using resting-state fMRI. Thirty healthy participants underwent resting-state fMRI, task fMRI, and then TMS hotspot localization. We found significant difference of locations between finger tapping fMRI activation and TMS hotspot. Specifically, the finger tapping fMRI activation was more lateral than the TMS hotspot in the premotor area. The fMRI activation peak and TMS hotspot were taken as seeds for resting-state functional connectivity analyses. Compared with TMS hotspot, finger tapping fMRI activation peak showed more intensive functional connectivity with, e.g., the bilateral premotor, insula, putamen, and right globus pallidus. The findings more intensive networks of finger tapping activation than TMS hotspot suggest that TMS treatment targeting on the fMRI activation area might result in more remote effects and would be more helpful for TMS treatment on movement disorders.

## Introduction

Transcranial magnetic stimulation (TMS) on the brain motor area has been utilized safely for decades in clinics for measuring the cortical excitability (Hanlon et al. 2015; Stinear et al. 2009), preoperative localization of motor function (Kallioniemi and Julkunen 2016; Pitkanen et al. 2015; Vitikainen et al. 2013), and repetitive TMS (rTMS) treatment for movement disorders (Wagle Shukla et al. 2016). The lateral motor cortices are among the most frequently used targets for the rTMS treatment of movement disorders, e.g., Parkinson’s disease (Wagle Shukla et al. 2016), stroke (Diekhoff-Krebs et al. 2017), tic disorder (Marsili et al. 2017), and writer’s cramp (Havrankova et al. 2010). Vast majority of these rTMS treatment studies used the hand motor hotspot as the stimulation target. The hand motor hotspot is defined as the location where the lowest intensity evoking the highest amplitude of motor evoked potential (MEP). Although the hotspot target is a way of individualized and precise localization, there has been no strong evidence that the hotspot is an effective target for any brain disorder. That is no wonder why rTMS has not been approved officially (e.g., by Food and Drug Administration in the USA) for the treatment of any movement disorder.

The brain activation area of a specific task (e.g., motor task) evaluated by functional magnetic resonance imaging (fMRI) has been taken as the stimulation target for the rTMS treatment in a few studies. For example, a study utilized a working memory task and defined fMRI activation in the dorsal lateral prefrontal cortex (DLPFC) as the stimulation target of rTMS treatment on multiple sclerosis (Hulst et al. 2017). However, few studies have used motor task fMRI activation as rTMS target in the lateral motor cortices. One reason might be that rTMS studies targeting on the motor cortices usually focused on movement disorder, and many patients with movement disorder have disability to perform motor task. Another possible reason is that the vast majority rTMS studies targeting on the hotspot claimed to stimulate the primary motor cortex (M1). These studies might have considered the hotspot and fMRI motor activation area the similar areas.

The primary motor area was structurally defined with the hand representation areas buried within the central sulcus and rarely extending to the gyral surface (Geyer et al. 1996). But the case is more complicated for fMRI motor activation studies and TMS hotspot studies. Many TMS studies considered the hotspot as the primary motor area (Benninger et al. 2011; Benninger et al. 2009; Khedr et al. 2003, 2006; Siebner et al. 2000) or hand knob (hand movement activation area, “knob-shaped” curvature of the precentral gyrus (Puce et al. 1995; Yousry et al. 1997)). However, an MRI-guided TMS study found that the hotspot was located at the pre-motor area, i.e., anterior to the hand knob (Ahdab et al. 2016). A few studies combined TMS and motor task fMRI and found that the hotspot was not overlapped with the motor task activation area (Bastings et al. 1998; Diekhoff et al. 2011; Herwig et al. 2002; Lotze et al. 2003b; Sparing et al. 2008).

The hotspot location is determined by MEP which reflects the conduction of the descending corticospinal or corticonuclear pyramidal tract (Lefaucheur 2019), while the fMRI activation reflects the hemodynamic changes when response to motor tasks (Bastings et al. 1998). The TMS and fMRI techniques provide related but different information. These two locations may have different underlying neural networks. Resting-state fMRI (RS-fMRI) seed-based functional connectivity is a systems-level approach to assess the relationship between the seed region and other brain areas. It represents the temporal synchronization of spontaneous brain activity among distributed brain regions associated with particular functions (Cole et al. 2010; Fox et al. 2005; Hampson et al. 2002; Van Dijk et al. 2010). A study successfully separated motor from sensory components by using functional connectivity, although the distance of these two cortices was very close (Zhang et al. 2009). RS-fMRI studies have shown that stimulating on the superficial target could affect the functional connectivity on remote brain areas (Eldaief et al. 2011; Wang et al. 2014) and hence improved memory performance (Wang et al. 2014).

The first aim of the current study was to explore the difference of the location of TMS hotspot and task fMRI activation in the precentral gyrus. More importantly, the second aim was to investigate the functional connectivity network differences of these locations. Since the finger tapping task required more active processing than TMS-induced finger movement, we hypothesized that the finger tapping activation area would have more intensive functional connectivity. If so, the results of the current study would help choose the stimulation target on the motor cortex for rTMS treatment of movement disorders.

## Materials and Methods

### Participants

Thirty right-handed healthy participants (16 females, 18–28 years old, mean age ± standard deviation: 22.9 y ± 2.7) were recruited by internet advertisement. All participants had no history of head trauma, substance abuse, or neuropsychiatric disorders. The whole study was carried out and approved by the Ethics Committee of the Center for Cognition and Brain Disorders (CCBD) at Hangzhou Normal University (HZNU). Informed consent was obtained from all individual participants included in the study.

### Experimental Design

Participants underwent a resting-state fMRI scan session and then a task fMRI session. Then they went to the TMS room.

In the resting-state fMRI session, participates were asked to keep their eyes closed, relax, remain as motionless as possible, not think of anything in particular, and not fall asleep. Foam pads were used to ensure the head comfortably and minimize the head motion.

**Fig. 1:**
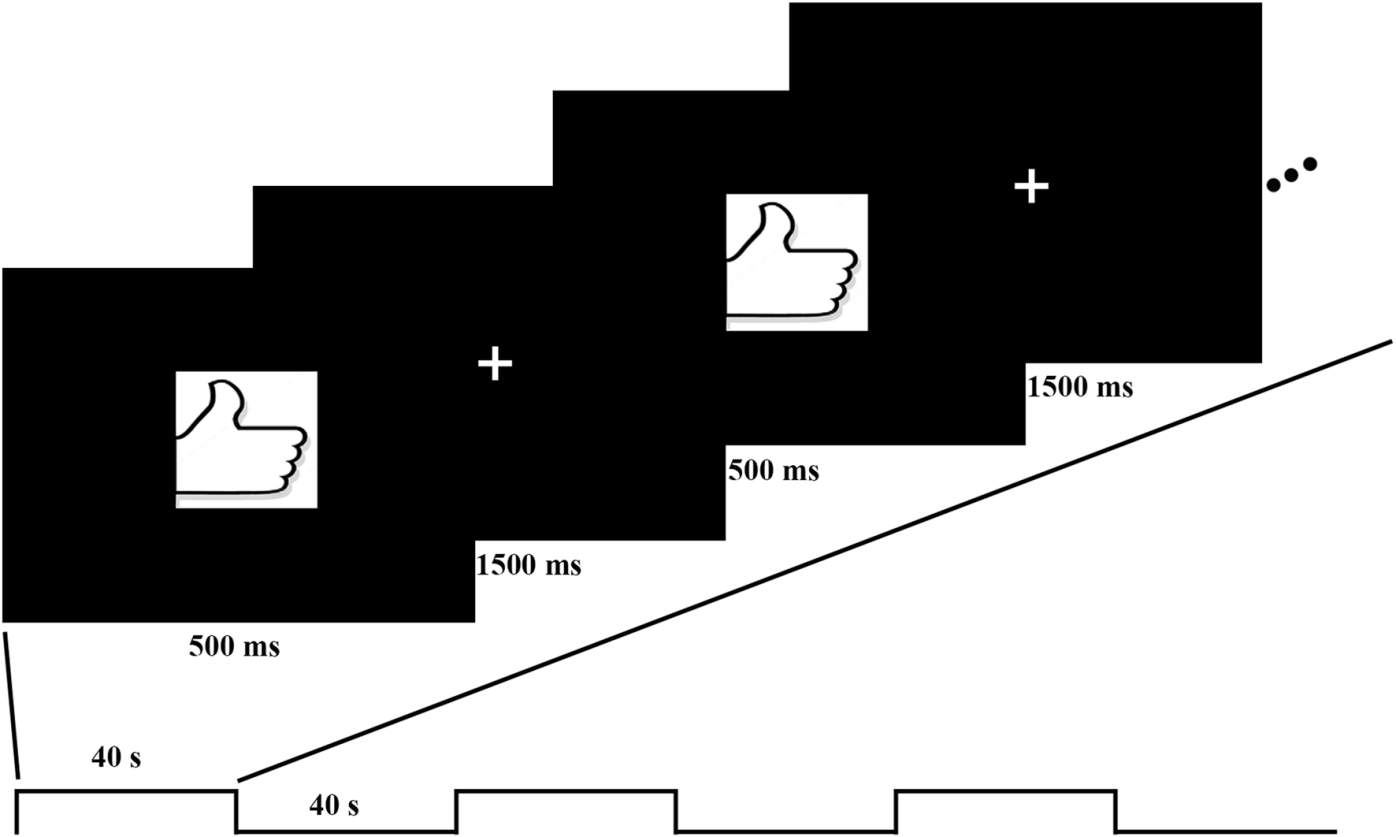
The block design task with right thumb tapping, during a 4-min scanning in MRI scanner

During the task fMRI session, participants were asked to perform a block design task of right thumb tapping during a 4-min scanning in MRI scanner. Participants placed their right hands palm uppermost and held a button in palm, then pressed the button with their right thumb when a picture of thumb appears in the center of the screen. The picture lasted 500 ms then followed a 1500-ms fixation. Each block lasted 40 s and there were 6 blocks in total (Fig. 1).

### Data Acquisition

#### MRI Data Acquisition

MRI data were acquired on a GE 3T scanner (MR-750, GE Medical Systems, Milwaukee, WI) at the Center for Cognition and Brain Disorders (CCBD) of Hangzhou normal university. The fMRI scanning sessions using gradient-echo echo planar imaging (EPI) sequences included an 8-min resting-state session and a 4-mintask session with the following parameters: repetition time (TR) = 2000 ms, echo time (TE) = 30 ms, flip angle (FA) = 90°, 43 slices with no gap, matrix = 64 × 64, field of view (FOV) = 220 × 220 mm, acquisition voxel size = 3.44 × 3.44 × 3.2 mm. A high resolution T1 anatomical image was scanned for accurate localization (176 sagittal slices, thickness = 1 mm, TR = 8.1 ms, TE = 3.1 ms, FA = 8°, FOV = 250 × 250 mm).

#### Electromyographic Recording

Surface electromyogram (EMG) was used to record the MEP amplitudes of the right abductor pollicis brevis (APB) muscle using 9-mm diameter Ag/AgCl surface electrodes with their centers 20-30 mm apart over the muscle bellies. The active electrode was placed over the venter musculi and the reference electrode was placed over metacarpophalangeal joint. The EMG signal of APB muscle was amplified (1000×), bandpass filtered (20 Hz–2.5 kHz; Intronix Technologies Model 2024F) and digitized at 5 kHz by an analog-to-digital interface (Micro 1401; Cambridge Electronics Design, Cambridge, UK), then saved in a computer and projected on a screen.

#### TMS Hotspot Measurement

Single pulse TMS (Magstim Co., Wales, UK) was employed with a figure-8 coil (diameter = 70 mm). Participants sat in a cozy chair with both arms relaxed on their thighs. Through visual and EMG monitoring, full muscle relaxation was ensured. The coil was firstly placed over the left M1 (hand knob) with the handle backwards and 45° against the sagittal midline of the brain for measuring the MEP in the target muscle. The coil orientation was monitored by BrainSight TMS navigation system (Rogue Research, Montreal, Canada) to keep it always perpendicular to the central sulcus and tangential to the scalp (Rossini et al. 2015). The hand motor hotspot was defined as the location around the “hand knob” where the lowest intensity evoking the highest amplitude of MEPs. To determine the hotspot, the coil was then shifted every 0.5 cm each time around the hand knob. The resting motor threshold (RMT) was quantified as the lowest intensity evoking a response (> 50 µV) in more than 5 of 10 consecutive trials. The stimulus intensity for all participants was lower than 50% of the maximal stimulator output (range 27 - 48%, mean ± stander deviation 38% ± 0.05). The number of stimuli for each position was maximal to 10. If the hotspot could not be determined within 10 stimuli, then the coil was moved to the next position. Finally, the hotspot of APB muscle was marked with BrainSight TMS navigation system on individual structure MRI images.

### Data Analyses

The fMRI data preprocessing of both task and resting-state was conducted by using DPABI_V3.0 (http://rfmri.org/dpabi) software (Yan et al. 2016).

#### Task fMRI Data Preprocessing

The task data preprocessing included the following steps: (1) correcting for the acquisition time delay between slices, (2) rigid-body realigning for estimation and correction of the motion displacement (all participants’ head motion less than 1 mm in translation or 1 degree in rotation in any direction), (3) co-registering the functional images to T1 image and then normalizing to MNI space using the echo-planar imaging (EPI) template in statistical parametric mapping 12 (SPM12, https://www.fil.ion.ucl.ac.uk/spm/software/spm12/), (4) spatial smoothing with a Gaussian kernel of 6-mm full width at half maxima (FWHM).

#### Resting-State fMRI Preprocessing

The resting-state fMRI data preprocessing included: (1) discarding the first 10 volumes to allow the signal to reach equilibrium and the subjects to adapt to the scanning noise, (2) correcting for the acquisition time delay between slices, (3) rigid-body realigning for estimation and correction of the motion displacement (all participants’ head motion less than 1 mm in translation or 1 degree in rotation in any direction), (4) normalizing to MNI space using the EPI template in SPM12, (5) regressing out nuisance signals (white matter, cerebrospinal fluid signals, and 24 head-motion parameters (Yan et al. 2013)), (6) removing the linear trend, (7) band-pass (0.01–0.08 Hz) filtering, and (8) smoothing with a Gaussian kernel of 6-mm FWHM.

### Statistical Analysis

#### Task fMRI Activation Detection

**Fig. 2:**
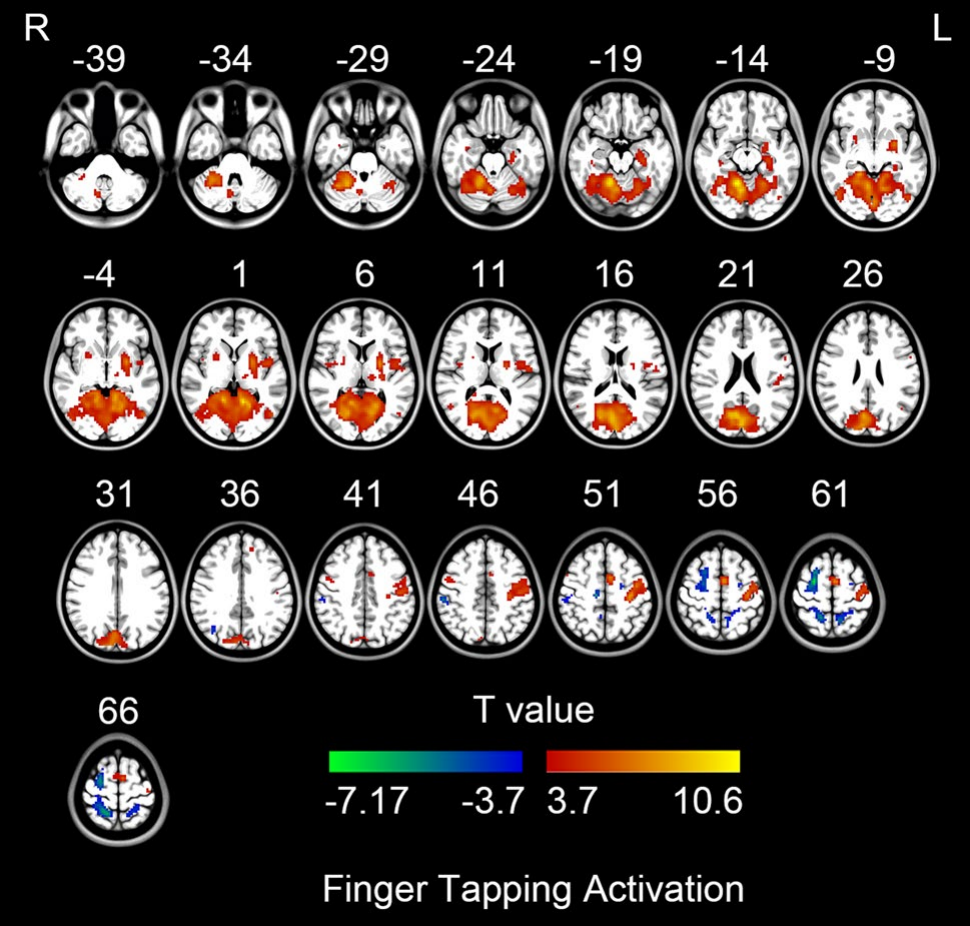
Group level finger tapping activation map (FDR correction, Q < 0.01). The warm color indicates the activation of finger tapping task; the cold color indicates the deactivation of finger tapping task. L, left hemisphere; R, right hemisphere

**Fig. 3:**
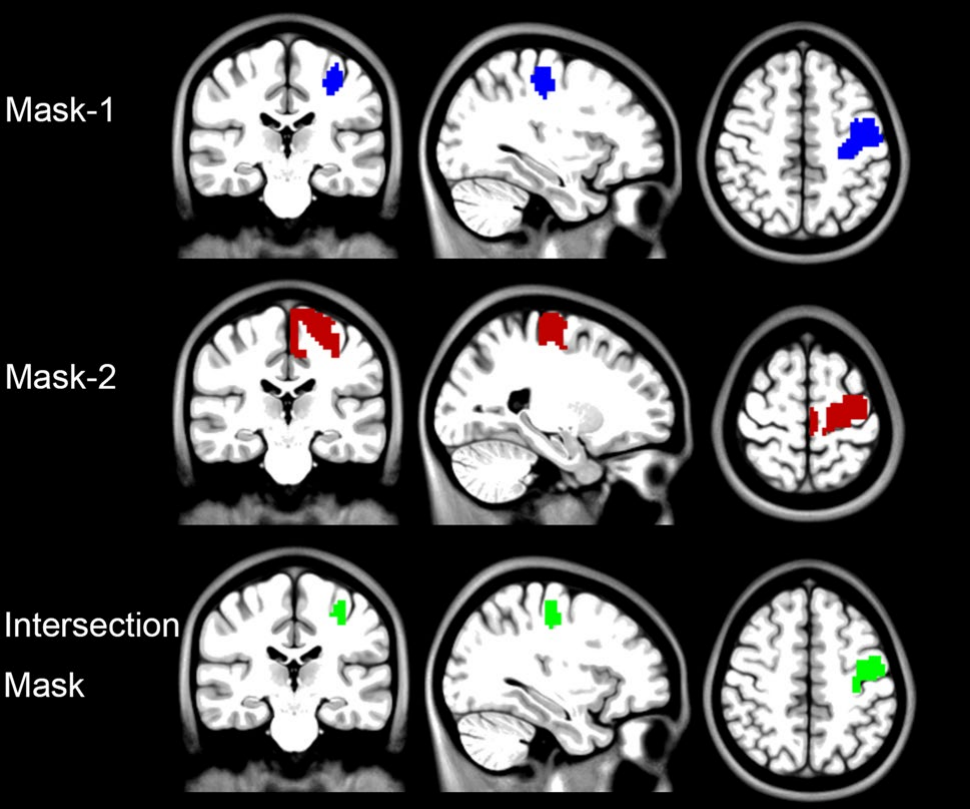
Generation of an intersection mask for the definition of the task activation peak voxel of each participant. The result of group level activation was saved as Mask-1, and the left precentral gyrus of Harvard-Oxford atlas was extracted as Mask-2. Then the intersection mask was generated by Mask-1 and Mask-2

SPM12 was used for subject-level activation analysis (high-pass filtering, > 1/128 Hz, was selected in “fMRI Model specification”) and group-level statistical analysis. The group-level activation map (Fig. 2, FDR correction, Q < 0.01) was saved as Mask-1. The left precentral gyrus of Harvard––Oxford atlas in FSL (http://www.fmrib.ox.ac.uk/fsl) was extracted as Mask-2. Then an intersection mask of Mask-1 and Mask-2 (Fig. 3) was generated to restrict the location of activation peak voxel of each participant.

#### Locations of Task fMRI vs. TMS Hotspot in the Precentral Gyrus

**Fig. 4:**
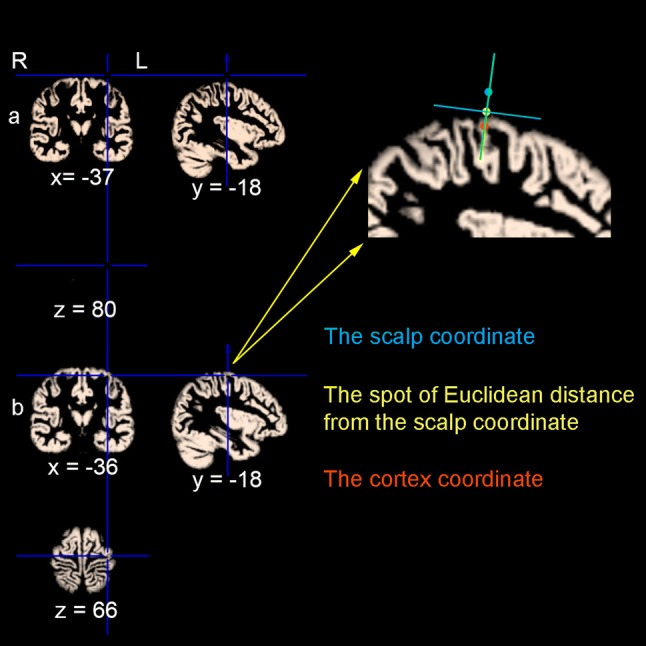
Diagram of the converting procedure from scalp coordinate to cortex coordinate (an example from a participant). The segmented spatially normalized structural image was thresholded with a grey matter probability > 0.2. **a** The MNI coordinate of hotspot for APB was exported from BrainSight system. **b** The converted cortex coordinate. The blue dot indicates the scalp coordinate, the yellow dot indicates the spot of Euclidean distance from the scalp coordinate, and the red dot indicates the cortex coordinate. The distance between yellow dot and red dot is 6 mm. The green line indicates the actual direction of coil which is perpendicular to the tangent (the blue line) passing through the spot

The peak voxel of each participant in the intersection mask was considered as the presentation of finger tapping task (Table 1). The MNI coordinates of hotspot for APB were exported from BrainSight system. It should be noted that these coordinates were all located above the scalp. We used a home-made toolkit TMStarget (http://www.brainhealthy.net) (Ji et al. 2017) to convert the exported scalp coordinates to cortex coordinates as follows. First, the structural images were segmented and spatially normalized by “new segment” in SPM12, and the normalized grey matter images were thresholded with a grey matter probability > 0.2. Second, the Euclidean distance between the scalp coordinates and the surface of cortices were estimated. Accordingly, the spot of Euclidean distance from the scalp coordinate was marked, and then went deep 6 mm further along the line which is perpendicular to the tangent passing through the spot. Finally, the location for each subject (Table 2) were determined as the presentation of TMS-induced movement of APB in the brain (Please see the Diagram of Fig. 4). These coordinates were employed to compare the difference of locations between fMRI finger tapping activation and TMS-induced movement by using SPSS (https://www.ibm.com/analytics/spss-statistics-software).

**Table 1:** The information of peak voxels of finger tapping activation in the intersection mask

Subject ID	Brodmann area	Coordinate (X Y Z)			T value	p Value (uncorrected)
Sub001	6	− 54	3	42	3.65	< 0.001
Sub002	6	− 48	− 3	51	5.87	< 0.001
Sub003	6	− 51	− 12	54	7.13	< 0.001
Sub004	4	− 51	− 12	45	5.28	< 0.001
Sub005	6	− 45	6	42	5.22	< 0.001
Sub006	4	− 39	− 18	54	3.80	< 0.001
Sub007	4	− 36	− 18	51	5.75	< 0.001
Sub008	4	− 42	− 18	51	4.33	< 0.001
Sub009	6	− 39	− 15	54	4.68	< 0.001
Sub010	4	− 51	− 9	45	6.49	< 0.001
Sub011	6	− 48	− 3	48	1.50	< 1
Sub012	6	− 48	− 3	51	4.90	< 0.001
Sub013	4	− 36	− 18	54	4.12	< 0.001
Sub014	6	− 42	− 6	57	6.53	< 0.001
Sub015	6	− 42	− 6	57	7.81	< 0.001
Sub016	4	− 42	− 18	54	8.07	< 0.001
Sub017	6	− 48	0	51	1.59	< 0.1
Sub018	6	− 36	− 12	54	6.09	< 0.001
Sub019	6	− 51	− 9	48	4.77	< 0.001
Sub020	6	− 54	− 3	45	3.65	< 0.001
Sub021	3	− 39	− 21	51	3.77	< 0.001
Sub022	4	− 39	− 21	54	8.28	< 0.001
Sub023	6	− 42	− 6	57	3.05	< 0.005
Sub024	6	− 42	− 3	48	3.90	< 0.001
Sub025	6	− 54	0	42	2.01	< 0.05
Sub026	4	− 51	− 12	45	5.59	< 0.001
Sub027	6	− 42	− 3	57	4.02	< 0.001
Sub028	6	− 36	− 15	60	7.22	< 0.001
Sub029	6	− 42	− 6	57	3.43	< 0.001
Sub030	4	− 48	− 12	42	4.49	< 0.001
Mean		− 45	− 9	51

**Table 2:** The coordinates of TMS APB hotspot

Subject ID	Brodmann area	Montreal Neurological Institute (X Y Z)		
Sub001	6	− 36	− 18	66
Sub002	6	− 24	− 18	69
Sub003	6	− 28	− 2	67
Sub004	4	− 47	− 18	59
Sub005	6	− 37	− 12	65
Sub006	6	− 40	− 6	63
Sub007	4	− 45	− 11	64
Sub008	6	− 43	0	56
Sub009	6	− 38	− 8	62
Sub010	4	− 38	− 19	65
Sub011	6	− 40	− 8	63
Sub012	6	− 33	− 7	66
Sub013	3	− 52	− 22	54
Sub014	6	− 50	− 4	55
Sub015	6	− 25	− 3	68
Sub016	6	− 18	− 6	69
Sub017	4	− 45	− 16	67
Sub018	6	− 41	− 11	65
Sub019	6	− 34	− 14	68
Sub020	6	− 35	− 15	64
Sub021	6	− 42	− 9	63
Sub022	4	− 42	− 14	66
Sub023	6	− 38	− 19	67
Sub024	6	− 31	− 12	68
Sub025	6	− 42	− 12	64
Sub026	6	− 28	− 14	70
Sub027	6	− 38	− 16	65
Sub028	6	− 19	− 18	71
Sub029	3	− 43	− 29	56
Sub030	6	− 46	− 12	60
Mean		− 37	− 12	64

The coordinate of Z axis was estimated. Please see the Materials and methods section, “Locations of task fMRI vs. TMS hotspot in the precentral gyrus” for details

*TMS* transcranial magnetic stimulation, *APB* abductor pollicis brevis

#### Resting-State fMRI Functional Connectivity

The coordinates of fMRI finger movement peak voxel in Table 1 and TMS-induced movement presentations in Table 2 were taken as seeds for computing voxel-wise functional connectivity of resting-state fMRI data. For each participant, the mean time course of a sphere (radius 4 mm) centered at the seed coordinate was acquired, then the functional connectivity was calculated. One-sample t-tests were performed at group level for each type of movement. Voxels above a corrected threshold (FDR correction, Q < 0.0001 and Q < 0.000000005) were taken as showing significant functional connectivity with the seed region. Paired t-tests were conducted between the two types of functional connectivity maps throughout the whole brain. Voxels above corrected threshold (GRF correction, single voxel p < 0.001, cluster level p < 0.05) were taken as showing significant different functional connectivity between the two types of movement.

## Results

### The Distance Between fMRI Finger Tapping Activations and TMS Hotspot

**Fig. 5:**
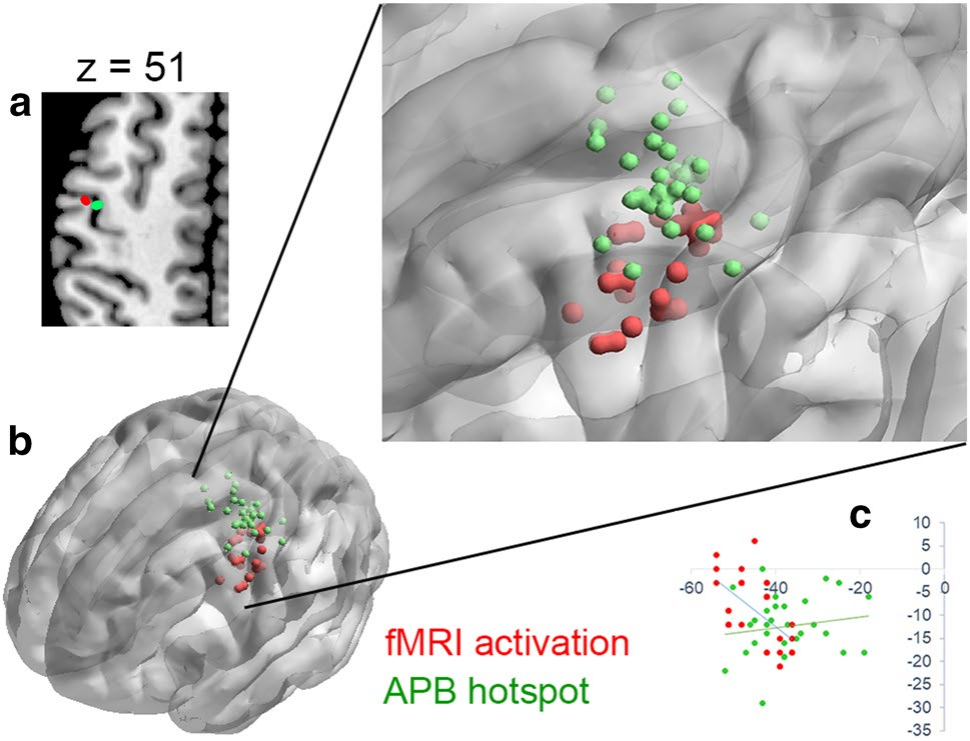
Localization of the premotor area with fMRI activation and TMS hotspot. **a** The red ellipsoid indicates mean MNI coordinates (± SD) of fMRI activation peak voxel, and the green ellipsoid indicates mean MNI coordinates (± SD) of the TMS hotspot. All these coordinates sat in left hemisphere. **b** Individual locations project to a three-dimensional brain template. **c** A scatter diagram of individual locations

Paired t-tests showed that there were significant differences in X and Z axes (Bonferroni correction, 0.05/3 = 0.0167, the actual p < 0.001, Table 3). The thumb tapping activations sat more laterally and more inferior (X and Z axes, respectively. Table 3). Both thumb tapping activation and TMS-induced movement representations were not at “hand knob” (Fig. 5).

**Table 3:** The differences of spatial localizations (finger tapping activation vs. APB hotspot)

	Mean coordinate (mm) ± standard deviation		
	x	y	z
Activation peak	− 44.6 ± 5.9	− 9.1 ± 7.4	50.7 ± 5.3
APB hotspot	− 37.3 ± 8.5	− 12.4 ± 6.5	64.2 ± 4.5
T value	− 3.7	1.7	− 10.5
p Value	0.001*	0.102	< 0.001*

*APB* abductor pollicis brevis

^*^Bonferroni correction

### Brain Areas of Finger Tapping Task fMRI Activation

The right thumb tapping task activated extensive brain areas including the bilateral putamen, bilateral insula, ipsilateral precentral gyrus, cerebellum, visual area, contralateral precentral gyrus, supplementary motor area, and thalamus (Fig. 2, Table 4).

**Table 4:** Finger tapping activations in the whole brain

Brain region	Brodmann area	Montreal Neurological Institute (X Y Z)			Cluster size (mm^3^)	T value	Q value
Right Occipital Lobe	19	12	− 54	− 15	126,198	10.60	< 0.01
Right Hippocampus	20	33	− 3	− 27	216	4.62	< 0.01
Left Putamen		− 24	− 3	6	13,554	7.40	< 0.01
Right Hippocampus	20	36	− 27	− 15	378	4.18	< 0.01
Right Putamen		24	6	0	1350	4.67	< 0.01
Left Thalamus		− 12	− 21	6	621	4.63	< 0.01
Right Insula	48	36	− 3	9	810	4.22	< 0.01
Right Lateral Occipital Cortex	37	51	− 60	12	189	4.45	< 0.01
Left Postcentral Gyrus	48	− 51	− 21	21	486	4.27	< 0.01
Left Precentral Gyrus	6	− 57	6	24	135	4.64	< 0.01
Left Angular Gyrus	39	− 48	− 66	24	162	3.98	< 0.01
Left Precentral Gyrus	4	− 33	− 21	48	9396	7.49	< 0.01
Left Frontal Pole	32	− 12	42	39	351	4.30	< 0.01
Left Supplementary Motor Area	6	− 6	0	54	3834	8.39	< 0.01
Right Precentral Gyrus	6	51	0	45	648	4.67	< 0.01

*Q value* false discovery rate (FDR) correction

### Functional Connectivity Results

**Fig. 6:**
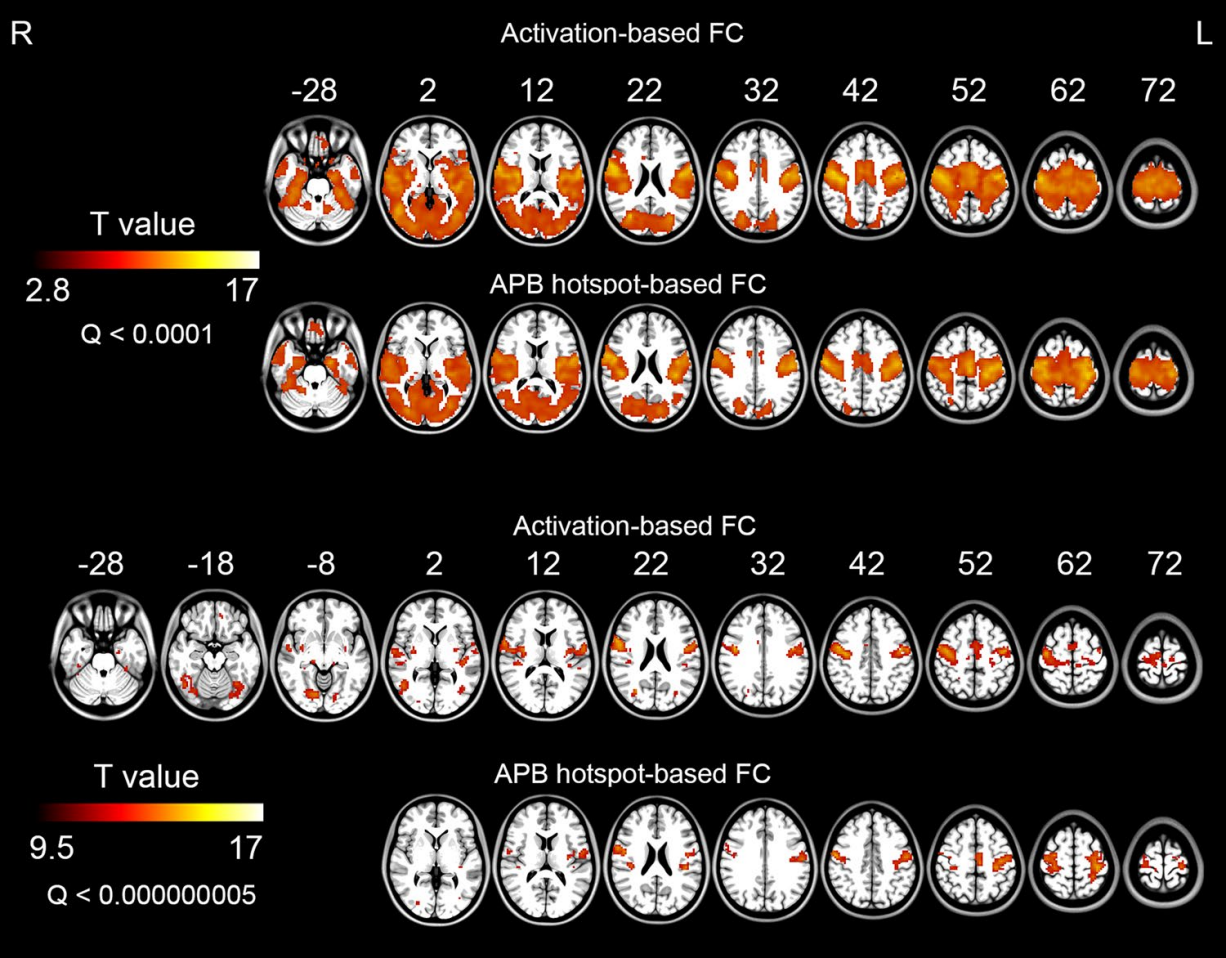
The activation-based and APB hotspot-based whole brain functional connectivity maps (FDR correction, Q < 0.0001 for upper row, Q < 0.000000005 for lower row). L, left hemisphere; R, right hemisphere; FC, functional connectivity

The activation-based and APB hotspot-based functional connectivity maps were shown in Fig. 6 (Fig. 6 upper row). Each of the two types of seeds showed extensive functional connectivity with the bilateral sensorimotor area, occipital area, cerebellum, and prefrontal gyrus. Their maps looked similar by visual inspection. Under a rigorous threshold, the different seeds showed very different functional connectivity patterns throughout the whole brain by visual inspection (Fig. 6 lower row).

**Fig. 7:**
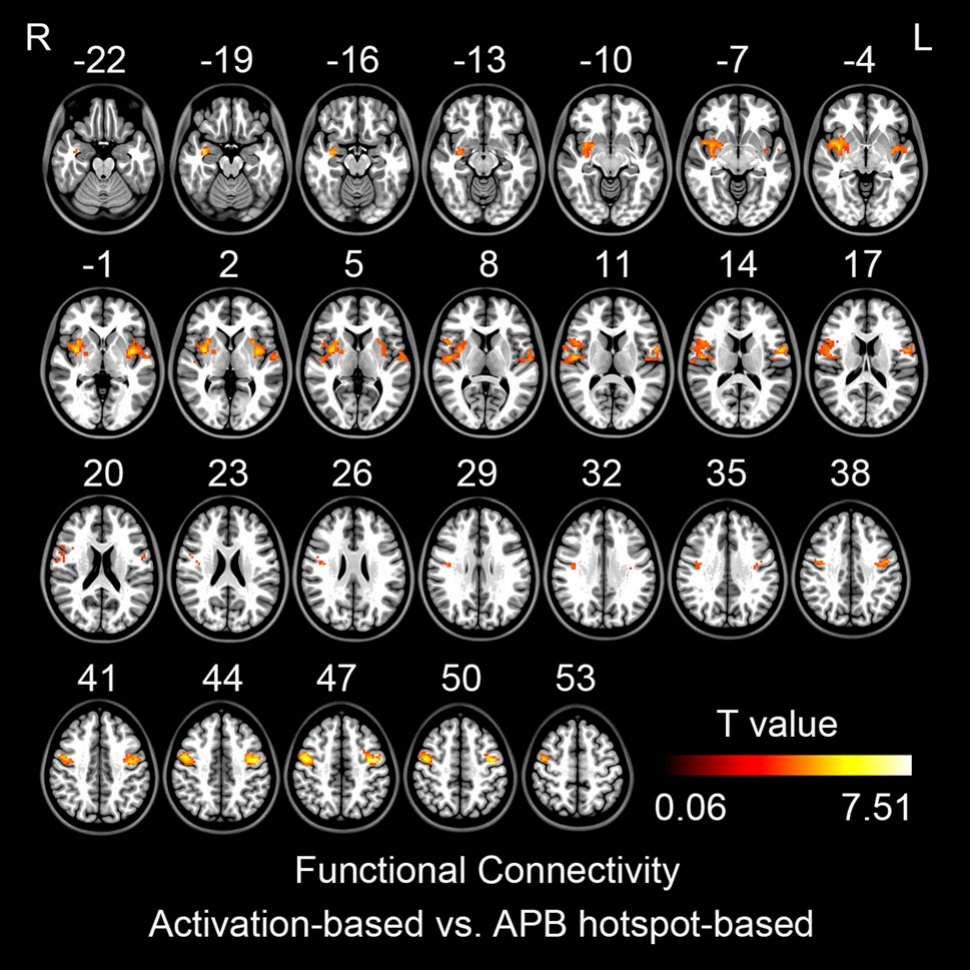
The differences between activation-based and APB hotspot-based functional connectivity (GRF correction, single voxel p < 0.001, cluster level p < 0.05). The warm color indicates more intensive activation-based functional connectivity than APB hotspot-based functional connectivity. L, left hemisphere; R, right hemisphere

Paired t-tests showed that the finger tapping activation seeds had significantly more intensive functional connectivity than TMS-induced seeds, including the right globus pallidus, bilateral putamen, bilateral insula, and bilateral precentral gyri (Fig. 7, Table 5). No any significantly more intensive functional connectivity was found for TMS-induced than that of finger tapping activation seed.

**Table 5:** The differences between activation-based and APB hotspot-based functional connectivity

Brain region	Brodmann area	Montreal Neurological Institute (X Y Z)			Cluster size (mm^3^)	T value	Peak voxel p value
Right Hippocampus (extend to right putamen, right globus pallidus and right insula)	20, 48	36	− 6	18	12,555	6.19	< 0.001
Left Insula (extend to left putamen)	48	− 39	0	0	5670	5.63	< 0.001
Right Precentral Gyrus	6	45	− 6	51	3969	6.33	< 0.001
Left Precentral Gyrus	6	− 45	− 6	48	3375	7.54	< 0.001

*APB* abductor pollicis brevis

## Discussion

### Different Locations of Finger Tapping fMRI Activation and TMS Hotspot

The current study found a spatial mismatch, i.e., more lateral fMRI activation than the hotspot, of the motor cortical locations between finger tapping fMRI activation and TMS-induced finger movement (hotspot), being consistent with the previous studies in which the authors also found the location of fMRI activation is lateral to the TMS hotspot (Diekhoff et al. 2011; Sparing et al. 2008). Moreover, some other studies found that the TMS hotspot location was more anterior to the fMRI activation (Bastings et al. 1998; Herwig et al. 2002; Lotze et al. 2003b). Many factors may have contributed such mismatch. The exact fMRI tasks varied across studies, such as the mode of movement (visual guided or self-paced), number of fingers (thumb movement solely or thumb movement against to each of the four other fingers, or “hand clenching”) (Bastings et al. 1998; Boroojerdi et al. 1999; Diekhoff et al. 2011; Herwig et al. 2002; Sparing et al. 2008). The parameters of TMS hotspot may vary across studies, e.g., the coil orientation. Although all these TMS studies placed the coil over the motor area with 45° against the middle line of brain with the handle backwards, the actual current orientation to the neurons highly depends on individual neuroanatomy and tissue anisotropy (Rossini et al. 2015). Such mismatch may also be due to a fact that the fMRI motor tasks are much more complicated than TMS-induced finger movement. Compared with TMS-induced finger movement, the finger tapping fMRI task involves more voluntary movement. A previous fMRI study compared voluntary movement and non-voluntary movement and found that the voluntary movement activation peak voxel sat more anteriorly and laterally in the contralateral primary motor area (Lotze et al. 2003a), being consistent with the present study (TMS-induced finger movement can be considered as non-voluntary movement). Therefore, the mismatch locations between finger tapping fMRI activation and TMS hotspot might be partly due to voluntary vs. non-voluntary movements. There might be also other components contributed to the location differences derived from visual cue that informed the participant to move. Non-human primate study found that a category of neurons responds to an object during observation without any movement, called visuomotor neurons (Raos et al. 2004). However, fMRI could not discern whether the activation of finger tapping task with a visual cue is contributed by this category of neurons. Even so, fMRI studies reported the premotor area responds to visual input, which codes information during the movement preparation (Gallivan et al. 2013). The premotor area involves in decoding of familiar objects and planning a motor action (Smith and Goodale 2015). fMRI activation location relates to more cognitive and voluntary elements of movement than TMS hotspot location.

### Different Functional Connectivity for Finger Tapping fMRI Activation and TMS Hotspot

We found that the finger tapping fMRI activation sat more laterally than TMS hotspot (Tables 1, 2 and 3). We were then interested in their difference of functional connectivity. Paired t-tests showed that the finger tapping activation seed had significantly more intensive functional connectivity than TMS hotspot seed. These brain areas included the bilateral putamen, right globus pallidus, bilateral insula, and bilateral premotor area (Fig. 7, Table 5). No any significant more intensive functional connectivity was found for the TMS hotspot seed than the finger tapping activation seed.

Premotor area has been widely reported to be involved in cognitive processing for movement. Motor preparation and execution are two stages of voluntary movements, involving M1, dorsal premotor cortex (PMd), ventral premotor (PMv) and supplementary motor area (Chouinard and Paus 2006; Gallivan et al. 2013, 2011a, b; Hirose et al. 2018; Nambu et al. 2015). A previous TMS study showed that, after low-frequency repetitive TMS applied over PMd, subjects could not select appropriate weight from two different weights to lift based on the visual cues (Chouinard et al. 2005). The current findings of more intensive functional connectivity of bilateral premotor areas with the finger tapping task activation than with TMS hotspot were in line with the comprehensive function of premotor area mentioned above.

Basal ganglia are key node in the motor circuits. The posterior (sensorimotor) putamen had been observed in finger movement task (Lehericy et al. 2005). And putamen was involved in the motor loop circuit (Middleton and Strick 2000). In our previous meta-analysis study, decreased amplitude low frequency fluctuations (ALFF) were observed in bilateral putamen in Parkinson’s disease patients (Wang et al. 2018). In the present study, the intensive functional connectivity was revealed at left posterior putamen, right posterior and middle putamen-globus pallidus with the activation seed (Fig. 6, Table 5). The current results suggest that the finger tapping activation area is more related to motor circuits than the TMS hotspot.

### Potential Applications of fMRI-Guided TMS Treatment

The present study found that the finger tapping activation and TMS hotspot located differently in the motor cortex. Moreover, the finger tapping activation peak had more intensive functional connectivity with bilateral premotor area, bilateral insula, bilateral putamen and right globus pallidus. RS-fMRI functional connectivity studies have helped understand the complex mechanism of rTMS modulation effect on the brain activity. Some studies reported that the rTMS could modulate the brain network or functional connectivity (Andoh et al. 2015; Chen et al. 2013; Cocchi et al. 2016; Cocchi et al. 2015; Eldaief et al. 2011; Halko et al. 2014; Ji et al. 2017; Nettekoven et al. 2014, 2015; Wang et al. 2014; Watanabe et al. 2014). The current results of more intensive functional connectivity of finger tapping activation area than the TMS hotspot may suggest that TMS treatment targeting on the fMRI activation area might result in more remote effects than targeting on the hotspot. Therefore, the fMRI activation might be a more ideal target for improvement of motor functions or treatment of movement disorders. To our knowledge, we conducted the first study to demonstrate the different brain networks of motor task fMRI activation vs. TMS hotspot area. One clinical importance of our findings is that rTMS treatment on movement disorders may take the motor task fMRI activation area, but not the TMS hotspot, as individual and precise stimulation target. Although the TMS hotspot is also an individualized and precise stimulation target, this target may have less widespread effect on remote brain regions, because TMS hotspot region has much less intensive brain network than motor fMRI task activation area.

## Conclusion

Finger tapping fMRI activation location differs from the TMS hotspot and has more intensive functional connectivity with motor-related brain regions, e.g., premotor area and basal ganglia. The current results might be of clinical importance for rTMS treatment target selection. Future rTMS studies could take the motor task fMRI activation area as individual and precise stimulation target for treatment on movement disorders.

## Limitations

A few limitations should be addressed. (1) Repetitive TMS was not performed, therefore, it is not known whether rTMS could differently modulate the hotspot network and motor task activation network; (2) We speculated that the hotspot may be more related to passive movement, however, due to the limitations of the experimental equipment, the present study did not measure the passive movement in fMRI. (3) It has been reported that the fMRI activation peak could be different if different fMRI imaging sequences were used (e.g., spin-echo vs. gradient-echo) (Diekhoff et al. 2011), future studies should take this issue into account.
